# Complementary medicine use in US adults with a history of colorectal cancer: a nationally representative survey

**DOI:** 10.1007/s00520-020-05494-x

**Published:** 2020-05-01

**Authors:** Charlene HL Wong, Tobias Sundberg, Vincent CH Chung, Petra Voiss, Holger Cramer

**Affiliations:** 1 Jockey Club School of Public Health and Primary Care, The Chinese University of Hong Kong, Hong Kong, China; 2 Musculoskeletal and Sports Injury Epidemiology Center (MUSIC), Institute of Environmental Medicine, Karolinska Institutet, Stockholm, Sweden; 3 Australian Research Centre in Complementary and Integrative Medicine (ARCCIM), Faculty of Health, University of Technology Sydney, Sydney, New South Wales Australia; 4 School of Chinese Medicine, The Chinese University of Hong Kong, Hong Kong, China; 5 Department of Internal and Integrative Medicine, Evang. Kliniken Essen-Mitte, Faculty of Medicine, University of Duisburg-Essen, Am Deimelsberg 34a, 45276 Essen, Germany

**Keywords:** Colorectal cancer, Complementary therapies, Mind-body medicine, Public health

## Abstract

**Background:**

In the USA, colorectal cancer is among the top diagnosed cancers. The current study specifically targets the US adult population that have a history of colorectal cancer.

**Methods:**

We used the 2017 National Health Interview Survey (NHIS) to investigate the prevalence and predictors of colorectal cancer survivors using complementary medicine in the past 12 months in a representative sample of the US population (*N* = 26,742). We descriptively analyzed the 12-month prevalence of any complementary medicine use separately for individuals with a prior diagnosis of colorectal cancer and those without. Using chi-squared tests and backward stepwise multiple logistic regression analyses, we identified predictors of complementary medicine use in the past 12 months.

**Results:**

A weighted total of 1,501,481 US adults (0.6%) had a history of colorectal cancer. More individuals without (weighted *n* = 76,550,503; 31.2%) than those with a history of colorectal cancer (weighted *n* = 410,086; 27.3%) had used complementary medicine. The most commonly used complementary medicine among colorectal cancer patients was mind-body medicine, followed by chiropractic. A higher prevalence of complementary medicine use was associated with being female, higher educated and/or living in the US Midwest or South.

**Conclusions:**

In this study, over one fourth of the US colorectal cancer survivors had used complementary medicine. Mind-body medicine was found to be the most commonly used. With evidence supporting the effectiveness and safety of mind-body medicine use among colorectal cancer patients, promoting the use of evidence-based mind-body medicine for colorectal cancer management could be considered.

## Introduction

The populations of North America, along with those in Europe, Australia, New Zealand, and Eastern Asia, have the highest age standardized incidence rates of colorectal cancer in the world [1]. In the USA, colorectal cancer is among the top diagnosed cancers [2], and in 2018, colorectal cancer represented 8.1% of all new cancer cases [3]. The lifetime risk of US men and women of developing colorectal cancer has been estimated to 4.2% [3]. It has been estimated that the global burden of colorectal cancer will grow by 60% over the next decade to encompass more than 2.2 million new cases and 1.1 million deaths by 2030 [4]. Considering the variation of the incidence of colorectal cancer between different regions and cultures, factors that may contribute to the expected increase are the economical and developmental changes in many low- and middle-income countries including the adoption of western lifestyle [4]. Similarly, it has been suggested that different lifestyle and dietary habits may be important factors for colorectal cancer prevalence [5, 6].

Current strategies and guidelines for colorectal diagnosis and treatment include screening and physician investigations to identify cancer pathology that if found can be treated both with local and systematic interventions such as surgery, chemotherapy, radiation therapy, and local ablation [7–10]. Of those being diagnosed with colorectal cancer in the USA, 64.5% are expected to survive 5 years or more after having received their diagnosis [3]. Albeit significant betterments in the treatment of patients with colorectal cancer and metastatic disease over the last decades [2], there are still significant burdens and suffering accompanying this disease. Living with colorectal cancer may relate to both physical and psychological health consequences including distress, depression, and bowel problems [11].

Living with colorectal cancer in the long-term typically involves continued conventional care and follow-ups to monitor cancer and medical health status. Recent research also supports the value of colorectal cancer patients to be physically active, which may lead to better quality of life [12]. It has also been reported that patients with colorectal cancer might complement their reliance of conventional care with other types of treatments and activities including the use of complementary medicines, whereby some research suggest that up to 75% of patients have used at least one complementary therapy [13]. Previous research targeting the US population report that 79% of cancer survivors use complementary medicine, and that those users were slightly more likely to be survivors of colorectal, breast, or melanoma cancers [14]. It was further reported that cancer patients’ main reasons for using complementary medicine was to support general wellness and pain and for cardiovascular reasons rather than for cancer specific concerns [14].

The current study specifically targets the US adult population that have a history of colorectal cancer and investigates the prevalence and predictors of them using complementary medicine.

## Methods

### Study design

We analyzed data from the 2017 US National Health Interview Survey (NHIS), a nationally representative interview survey monitoring the health of the non-institutionalized US population. More information on survey composition, sampling strategy, and administration of the NHIS can be found online [15]. A total of 32,617 households were included in the survey and 26,742 adults provided data (response rate: 80.7%) [16].

Specifically, we used data from the NHIS Person File and NHIS Sample Adult File. Prior cancer diagnoses were queried as follows: “Have you ever been told by a doctor or other health professional that you had cancer or a malignancy of any kind?” If this question was answered in the affirmative, the participant was asked “What kind of cancer was it?” Up to three different kinds of cancer were collected. We considered all participants who indicated that they had been told to have had colon cancer and/or rectal cancer as having received a diagnosis of colorectal cancer. We further included data on time since diagnosis (operationalized as age at time of the survey minus age at first diagnosis) as well as the socio-demographic characteristics age, sex, ethnicity, region, marital status, education, and employment. We defined complementary medicine use as having consulted with chiropractors, naturopaths, practitioners of chelation therapy, practitioners of traditional medicine, and/or homeopaths in the past 12 months and/or having used mind-body medicine approaches in the past 12 months. We considered the use of mantra meditation, mindfulness meditation, spiritual meditation, guided imagery, progressive relaxation, yoga, tai chi, and/or qi gong as having used mind-body medicine.

### Statistical analysis

We descriptively analyzed the 12-month prevalence of any complementary medicine use separately for individuals with a prior diagnosis of colorectal cancer and those without such a diagnosis. We further analyzed the 12-month prevalence of consultations chiropractors, naturopaths, practitioners of chelation therapy, practitioners of traditional medicine, homeopaths, and/or of the use mind-body medicine approaches. Since the NHIS oversamples minorities, we calculated population-based estimates using weights calibrated to the 2010 census-based population estimates for age, gender, and ethnicity of the US civilian non-institutionalized population.

Using chi-squared tests, we compared socio-demographic and clinical characteristics between (a) individuals with versus those without a prior diagnosis of colorectal cancer, and (b) individuals with a prior diagnosis of colorectal cancer who had used versus those who had not used complementary medicine in the past 12 months. We included the following independent variables in the analysis: age (categories: 18–29, 30–39, 40–49, 50–64, 65 years or older), ethnicity (categories: non-Hispanic White, Hispanic, African American, Asian, Other), region (categories: West, Northeast, Midwest, South), marital status (categories: not in relationship; in relationship), education (categories: less than college, some college, or more), employment (categories: employed, unemployed), and time since cancer diagnosis (categories: up to 1 year, 2–5 years, more than 5 years).

To analyze independent predictors of complementary medicine use in the past 12 months, we utilized backward stepwise multiple logistic regression analyses. Including too many potential predictor variables in the analysis can dilute true associations due to wide confidence intervals or identify spurious associations [17]. We therefore only considered those potential predictors associated with mind-body medicine use in univariate analysis (chi-squared test) for the multivariate analysis. The cutoff for significance in univariate analysis was chosen more liberal (*p* value of ≤ 0.10) than common since its purpose was to identify potential predictor variables rather than to test a hypothesis [17]. In the multivariate analysis, we calculated adjusted odds ratios with 95% confidence intervals (CI) and *P* values using relative weights and considered *P* values of ≤ 0.05 statistically significant in regression analysis. We used the Statistical Package for Social Sciences (IBM SPSS Statistics for Windows, release 25.0. Armonk, NY: IBM Corp.) for all analyses.

## Results

**Fig. 1:**
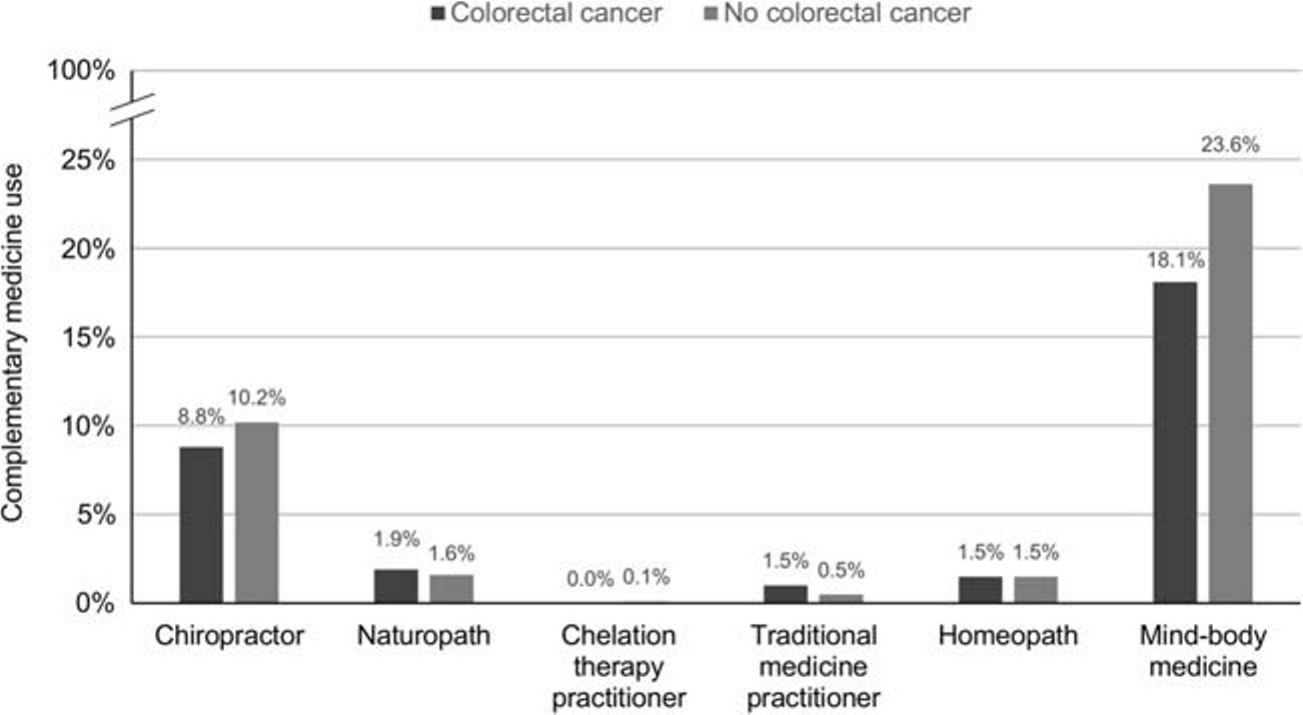
12-month prevalence of consultations with complementary medicine practitioners and of mind-body medicine use in individuals with and without a history of colorectal cancer. Weighted frequencies were used

We found that a weighted total of 1,501,481 US adults (0.6%) had a history of colorectal cancer. More individuals without (weighted *n* = 76,550,503; 31.2%) than those with a history of colorectal cancer (weighted *n* = 410,086; 27.3%) had used complementary medicine. In detail, the 12-month prevalence of consultations with a chiropractor and of using mind-body medicine was higher in individuals without a diagnosis (Fig. 1). The prevalence of consultations with naturopaths, practitioners of traditional medicine was higher in those with a prior colorectal cancer diagnosis (Fig. 1). Besides complementary medicine use, individuals with and without a history of colorectal cancer also differed on several socio-demographic characteristics (Table 1).

**Table 1:** Comparison of characteristics in individuals with and without a prior colorectal cancer diagnosis. Weighted frequencies are reported; *P* values are derived from chi-squared tests using relative weights

Characteristics	Individuals without colorectal cancer (weighted *n* = 245,155,790)	Individuals with colorectal cancer (weighted *n* = 1,501,481)	*P* values
Age			< 0.001
18 to 29 years	51,911,205 (21.1%)	0 (0.0%)	
30 to 39 years	42,100,721 (17.2%)	113,879 (7.6%)	
40 to 49 years	39,632,186 (16.2%)	64,613 (4.3%)	
50 to 64 years	62,818,950 (25.6%)	488,732 (32.5%)	
65 years and up	48,692,728 (19.9%)	834,257 (55.6%)	
Sex			0.052
Male	118,152,291 (48.2%)	837,797 (55.8%)	
Female	127,003,499 (51.8%)	663,684 (44.2%)	
Ethnicity			< 0.001
Non-Hispanic White	158,136,596 (64.5%)	1,192,554 (79.4%)	
Hispanic	39,280,118 (16.0%)	135,625 (9.0%)	
Black	30,113,985 (12.3%)	72,252 (4.8%)	
Asian	14,840,605 (6.1%)	99,141 (6.6%)	
Other	2,784,486 (1.1%)	1909 (0.1%)	
Region			< 0.001
West	58,127,572 (23.7%)	249,900 (16.6%)	
Northeast	44,723,365 (18.2%)	445,695 (29.7%)	
Midwest	53,423,558 (21.8%)	369,211 (24.6%)	
South	88,881,295 (36.3%)	436,675 (29.1%)	
Employment			< 0.001
Unemployed	91,334,035 (37.3%)	935,471 (62.3%)	
Employed	153,745,086 (62.7%)	566,010 (37.7%)	
Education			0.405
Less than college	88,094,863 (35.9%)	488,434 (32.5%)	
Some college or more	156,076,430 (63.7%)	992,350 (66.1%)	
Marital status			< 0.001
Not in a relationship	97,396,032 (39.7%)	404,582 (26.9%)	
In a relationship	147,382,833 (60.1%)	1,096,899 (73.1%)	

In univariate analysis, individuals with a prior colorectal cancer diagnosis using complementary medicine were more likely female, higher educated and/or living in the US Midwest or South than those not using complementary medicine (Table 2). In regression analyses, we found that independent predictors of complementary medicine use in individuals with a prior diagnosis of colorectal cancer included the following: women had 4.15 times the odds (95% CI 1.94 to 8.85; *p* < 0.001) of using complementary medicine compared to men, and individuals with at least some college education had 2.78 times the odds (95% CI 1.18 to 6.54; *p* = 0.020) compared with less educated individuals.

**Table 2:** Comparison of characteristics in individuals with a prior colorectal cancer diagnosis using or not using complementary medicine. Weighted frequencies are reported; *P* values are derived from chi-squared tests using relative weights

Characteristics	Not using complementary medicine (weighted *n* = 1,091,395)	Using complementary medicine (weighted *n* = 410,086)	*P* values
Age			0.159
18 to 29 years	0 (0.0%)	0 (0.0%)	
30 to 39 years	69,678 (6.4%)	4.4201 (10.8%)	
40 to 49 years	26,533 (2.4%)	38,080 (9.3%)	
50 to 64 years	345,996 (31.7%)	142,736 (34.8%)	
65 years and up	649,188 (59.5%)	185,069 (45.1%)	
Sex			< 0.001
Male	703,785 (64.5%)	134,012 (32.7%)	
Female	387,610 (35.5%)	276,074 (67.3%)	
Ethnicity			0.090
Non-Hispanic White	867,363 (79.5%)	325,191 (79.3%)	
Hispanic	68,115 (6.2%)	67,510 (16.5%)	
Black	59,807 (5.5%)	12,445 (3.0%)	
Asian	96,110 (8.8%)	3031 (0.7%)	
Other	0 (0.0%)	1909 (0.5%)	
Region			0.038
West	160,401 (14.7%)	89,499 (21.8%)	
Northeast	388,971 (35.6%)	56,724 (13.8%)	
Midwest	235,449 (21.6%)	133,762 (32.6%)	
South	306,574 (28.1%)	130,101 (31.7%)	
Education			0.039
Less than college	401,486 (36.8%)	869.48 (21.2%)	
Some college or more	669,212 (61.3%)	323,138 (78.8%)	
Employment			0.875
Unemployed	685,925 (62.8%)	249,546 (60.9%)	
Employed	405,470 (37.2%)	160,540 (39.1%)	
Marital status			0.399
Not in a relationship	274,922 (25.2%)	129,660 (31.6%)	
In a relationship	816,473 (74.8%)	280,426 (68.4%)	
Years since diagnosis			0.198
Up to 1 year	255,552 (23.4%)	49,800 (12.1%)	
2–5 years	271,318 (24.9%)	97,826 (23.9%)	
More than 5 years	561,181 (51.4%)	262,460 (64.0%)	

## Discussion

### Summary of findings

In this nationally representative interview survey, there was 0.6% of the US adults who reported a prior colorectal cancer diagnosis, and 27.3% of these cancer patients had used complementary medicine. The most commonly used complementary medicine among colorectal cancer patients was mind-body medicine, followed by chiropractic services. However, their prevalence was even higher in individuals without a diagnosis. While the prevalence of consultations with naturopaths, practitioners of traditional medicine was higher in those with a prior colorectal cancer diagnosis, these analyses were based on less than 10 individuals with a diagnosis and are such prone to bias.

Furthermore, being female, having higher education level and living in the US Midwest or South were associated with an increased consumption of complementary medicine. The predictors of their complementary medicine use were female gender and higher education level.

### Use of complementary medicine among colorectal cancer patients in other Western countries

While our nationally representative interview survey indicated that 27.3% of colorectal cancer patients in the USA had used complementary medicine, a former literature review showed that approximately 75% of colorectal cancer patients in Europe and Canada consumed at least one complementary medicine approach to improve their general health and physical well-being [13].

A Canadian survey demonstrated that patients’ use of complementary medicine increased dramatically following their colorectal cancer diagnosis as they would like to improve their bodies’ capacity to fight cancer [18]. Another survey indicated that the majority of colorectal cancer patients in Europe felt satisfied with the use of complementary medicine [19]. They believed that complementary medicine may bring them benefits in psychosocial functioning, positive effects in their empowerment, and direct involvement in their cancer care [20]. Other possible reasons for patients to use complementary medicine included patients’ intention to try each available treatment option and less concerns on the safety of complementary medicine modalities than conventional treatments [21].

### Predictors of the use of complementary medicine among colorectal cancer patients

Our findings were in line with previous studies on the complementary medicine use for managing cancer symptoms [22, 23], which showed that female gender and higher education level were predictors of using complementary medicine among colorectal cancer patients. These two predictors were also found to be associated with an increasing consumption of complementary medicine among patients in our study.

A certain number of psychological factors were found to be related to the use of complementary medicine among female colorectal cancer patients, namely, worrying of cancer recurrence, vigor, anger, and emotional distress [24]. As complementary medicine was suggested to be effective in improving psychosocial functioning [25], female patients may be more likely to consume it due to this potential benefit. Besides, it is noteworthy that cancer patients with higher education level may have an inherent negative attitude towards conventional medicine, as well as higher awareness of complementary medicine modalities [26]. These factors may account for colorectal cancer patients’ choice for complementary medicine.

### Potential risk of non-disclosure of complementary medicine use to physicians

Since the non-disclosure of complementary medicine use to physicians may possibly lead to drug interactions and adverse interactions with conventional pharmaceutical treatments [27, 28] among colorectal cancer patients, it is important to strengthen physicians’ knowledge about complementary medicine and their communications with patients [18, 29]. This may enhance the mutual trust between physicians and patients and increase their abilities on healthcare decision-making [30, 31].

A survey in Denmark demonstrated that over half of the colorectal cancer patients failed to disclose their consumption of complementary medicine to their physicians, and less than 10% reported to have been asked by their physicians about the use of complementary medicine [32]. In addition, non-disclosure of complementary medicine use is also common among cancer patients in the USA. The 2012 US NHIS also indicated that about 30% of cancer patients who consumed complementary medicine did not disclose the use of complementary medicine to their physicians [33]. The most frequently reported reasons for their non-disclosure of complementary medicine use were (i) the physician did not ask and (ii) the patients did not think that their physicians needed to know [33].

In our survey, the colorectal cancer patients’ disclosure of complementary medicine use to physicians was not assessed. Future rounds of NHIS may investigate the communications between physicians and colorectal cancer patients about the consumption of complementary medicine. The findings would inform the development of effective strategies to enhance patients’ disclosure of complementary medicine use and reduce the risk of adverse effects among patients [29].

### Mind-body medicine as the most commonly used complementary medicine for managing colorectal cancer

Findings of our study concur with those of the previous literature review [13] that mind-body medicine was one of the most commonly used form of complementary medicine modality among colorectal cancer patients. Since it is not uncommon for cancer patients to experience symptoms such as anxiety, pain, fatigue, and decreased quality of life, mind-body medicine which aims to use one’s mind to improve physical function and enhance health are becoming popular among cancer patients [34].

Existing evidence supports the effectiveness of different mind-body medicine modalities among colorectal cancer patients. For instance, mindfulness meditation and progressive relaxation showed significant effects in relieving stress [35, 36], while yoga and qigong are effective in reducing anxiety and improving sleep quality [37, 38]. Besides, qigong showed benefits in reducing fatigue, improving physical activity, and quality of life [39]. These mind-body modalities are safe in general, as long as they are practiced under the guidance from qualified instructors [40, 41].

Promoting rational use of evidence-based mind-body medicine in the local colorectal cancer community could therefore be considered. Future NHIS may investigate reasons and expenditure for mind-body medicine use among the US patients. This will identify patients’ possible clinical and biopsychosocial needs, as well as costs on consuming mind-body medicine. The promotion strategies for mind-body medicine among colorectal cancer patients will then be facilitated accordingly.

### Strengths and limitations

There are some strengths and limitations in this study. Since the 2017 NHIS focused on the US nationally representative sample of the population, it provided a robust epidemiological basis for investigating the patients’ characteristics and predictors on the consumption of healthcare services. This study offers critical insight into the prevalence, patterns, and predictors of complementary medicine use among colorectal cancer patients. Moreover, our findings may inform research funders to allocate resources on various research projects related to complementary medicine use, particularly on mind-body medicine, among these cancer patients.

Nonetheless, this is a cross-sectional study which only shows the associations between the use of complementary medicine and patients’ characteristics without examining the casual relationship. Our study is a secondary analysis of the existing data. Reasons for the use of complementary medicine as well as satisfaction with the complementary treatments among colorectal cancer patients should be assessed in the future NHIS to enhance comprehensiveness of the analysis. As the NHIS depends on retrospective self-reported data, the patients may possibly have recall bias regarding their complementary medicine use.

## Conclusion

In this study, over one fourth of the US colorectal cancer patients had consumed complementary medicine. Among different types of complementary medicine, mind-body medicine was found to be the most commonly used. With evidence supporting the effectiveness and safety of mind-body medicine use among colorectal cancer patients, promoting the use of evidence-based mind-body medicine for colorectal cancer management could be considered.

## Data Availability

The datasets analyzed during the current study are available from the National Center for Health Statistics (NCHS), https://www.cdc.gov/nchs/nhis/nhis_2017_data_release.htm
